# Experiment on monitoring leakage of landfill leachate by parallel potentiometric monitoring method

**DOI:** 10.1038/s41598-022-24352-w

**Published:** 2022-11-28

**Authors:** Xinmin Hu, Yalu Han, Yong Wang, Xiaopei Zhang, Lizhi Du

**Affiliations:** College of Construction Engineering, Jilin University, Changchun, 130026 China

**Keywords:** Urban ecology, Geophysics

## Abstract

The accumulation of municipal solid waste (MSW) in landfills often becomes a serious pollution source of geological environment and groundwater. The geological environment is the carrier of the landfill, and also the main pollution object of the landfill. The main pollution modes of the landfill site to the surrounding geological environment are purging, flushing, leachate, etc. If the leachate leakage cannot be found and repaired in time, it will cause serious harm to the geological environment and groundwater. The cost of geological environment and groundwater sampling through borehole surveys is high. Therefore, monitoring the seepage path and migration law of leachate is of great significance for determining the pollution range of the landfill site. In this study, by adjusting the grids of different sizes and changing the flow rate of leachate, the monitoring of fluid migration of different types of leachate was strengthened. The results show that the parallel potential monitoring method can quickly reflect the location and number of leachate points and the migration law of leachate. It provides effective reference data for landfill leachate monitoring.

## Introduction

The pollution of landfill leachate to surface water and ground water is mainly caused by the fermentation and erosion of rainwater. As one of the important sources of water supply, groundwater should be protected^1^. Among them, the pollution source caused by rapid urban development has become the main component of garbage leachate, including micro plastics, sludge and heavy metals, which have potential risks to the geological environment and groundwater^2–4^. The effective measures to ensure the normal operation of the landfill site are to establish a leachate collection and removal system and observe the impact of leachate on the mechanical properties of concrete^5–8^. It is necessary to monitor the leachate to prevent further deterioration of the surrounding environment of the landfill^9–11^.

Common ways to reduce the concentration of leachate include using vertical and horizontal wells in landfills^12,13^. The use of water level wells or pressure wells to monitor the falling distance of seepage from vertical and horizontal wells still has disadvantages such as low monitoring efficiency, discrete data and other interference^14,15^.

Parallel potential monitoring method is an efficient geophysical detection technology. At present, it is widely used in the field of monitoring the distribution and movement of landfill leachate and gas. It is found that detecting the resistivity value of the site can determine the leachate injection plume, positioning depth and transverse extension^16–19^. The monitoring study of water injection reservoir further reflects the resistivity variation characteristics of different media layer^20^. The potential value of waste area is affected by moisture content^21,22^. Borehole-to-surface method has a good effect on the identification of the boundary of resistance abnormal body and the monitoring of its migration law^23,24^. The location and extension path of landfill gas are located by electrical resistivity tomography (ERT)^25,26^. It is found that there is a certain correlation between the resistivity ρ and the volumetric moisture content θ in the experimental test, which is well expressed by Archie's law^22,27,28^. Time-lapse ERT is used to monitor the leachate plume^29–32^.

**Figure 1:**
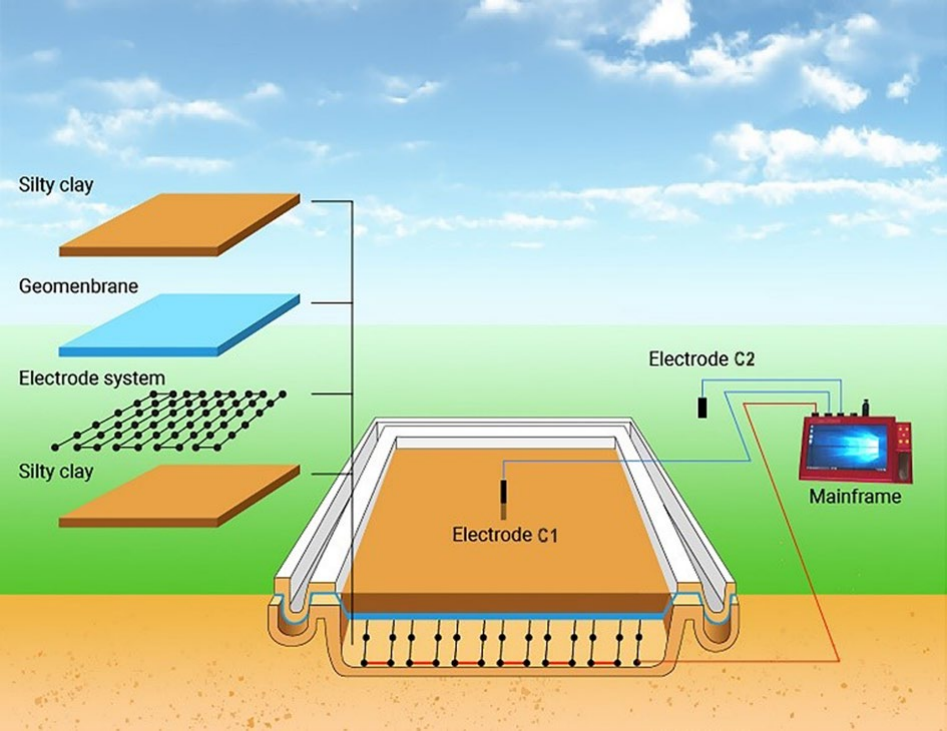
Schematic diagram of parallel potentiometric monitoring system.

A large number of experiments have been carried out on the monitoring of leachate migration by ERT method, which proves that this method has an obvious effect on monitoring the distribution of landfill leachate ^27,33–40^. Figure 1 shows that a parallel potential monitoring system is used to monitor leakage migration in the landfill lining system. The soil resistivity within a certain depth under the geomembrane is compared with the background field measured when the landfill is completed. The location and quantity of leakage and the morphological characteristics of plume are determined by this method.

## Materials and method

### Simulation experimental set up

Laboratory monitoring of leakage migration process can provide an important basis for field tests. The designed and improved ERT device can better describe the migration range of leakage in soil^41^. In this experiment, a parallel potential monitoring device was used to improve the monitoring of leakage fluid migration. The simulation experiment in the laboratory is carried out in a (100 cm*100 cm*50 cm) plexiglass tank. Sand and clay shall be screened with a 2.36 mm square sieve, watered and compacted with a board to ensure that the soil layer is in close contact with the measuring electrode.

### Electrode arrangement

**Figure 2:**
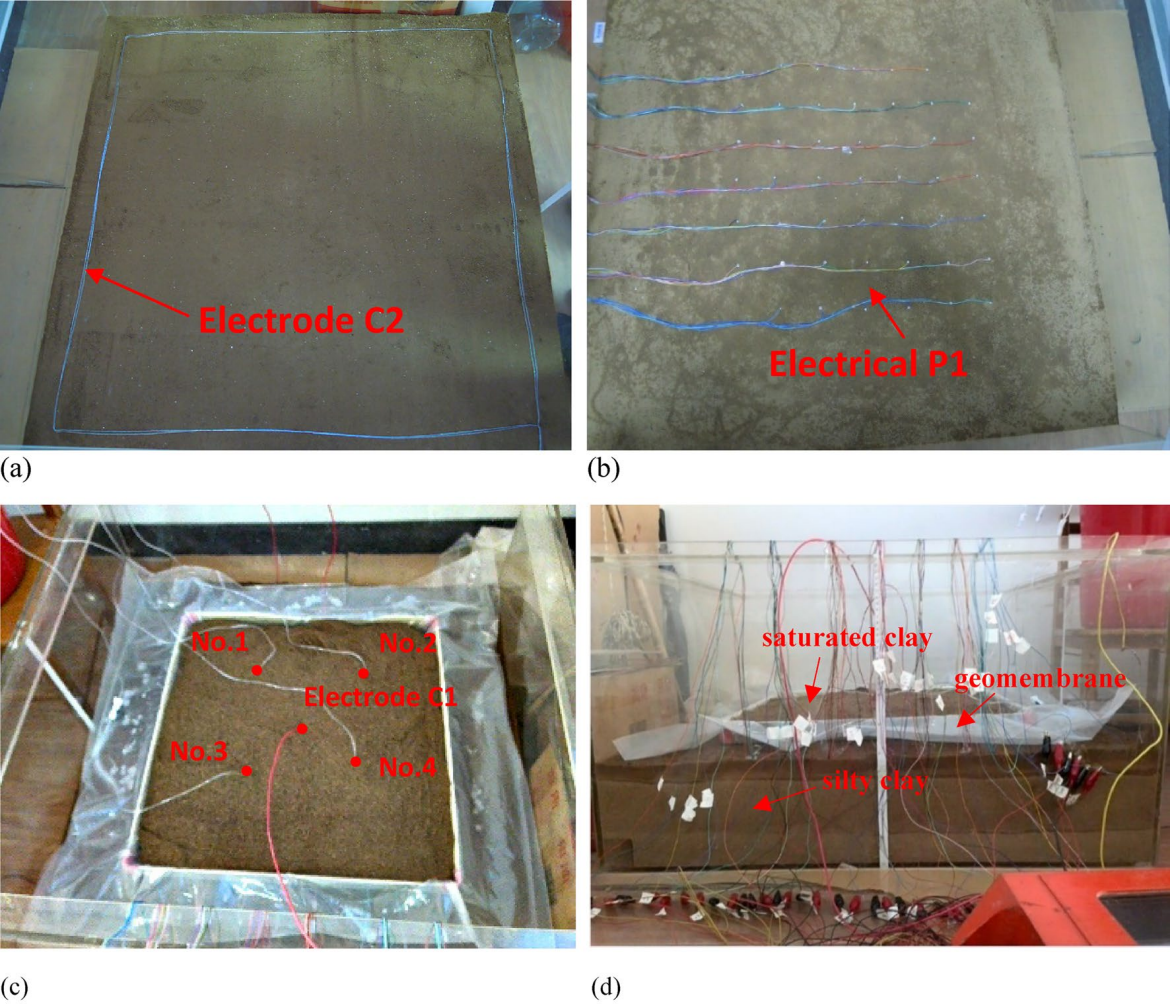
Set-up of leachate migration simulation experiment: (**a**) Schematic diagram of electrode C2 layout; (**b**) Schematic diagram of electrical system laying; (**c**) Position of electrode C1 and leakage point; (**d**) Schematic diagram of simulated experimental soil layer.

The ground wire of high-density electrical method instrument is connected to the electrodes arranged around the bottom of the tank as the power electrode C2, as shown in Fig. 2a. The host is connected to the electrode system. The electrode system consists of 47 electrode grids with a spacing of 0.08 m. The measuring electrode P1 is connected to the mainframe through a wire 0.05 m below the grid center. The geomembrane is located 0.03 m above the measuring electrode P1. The collection device is used as a monitoring system for various leachate. The arrangement of electrodes is shown in Fig. 2b. The power supply electrode C1 is placed at a certain depth in the middle of the saturated sand to provide a constant current. The location of electrode C1 and leakage point is shown in Fig. 2c. The layers from the bottom of the tank are silty clay, geomembrane, silty clay and saturated sand, as shown in Fig. 2d.

### Composition of monitoring system

The electrode system is used to monitor the background electric field and artificial electric field of the landfill site. In the experiment, the electrode system is laid in the clay layer under the geomembrane. It is composed of detection electrodes distributed in a grid at a certain distance.

The electrical signal conversion system adjusts the measurement mode, sampling accuracy, acquisition frequency and other parameters of the electrode in the field according to the instructions of the mainframe, and transmits the collected electrical signal to the mainframe.

**Figure 3:**
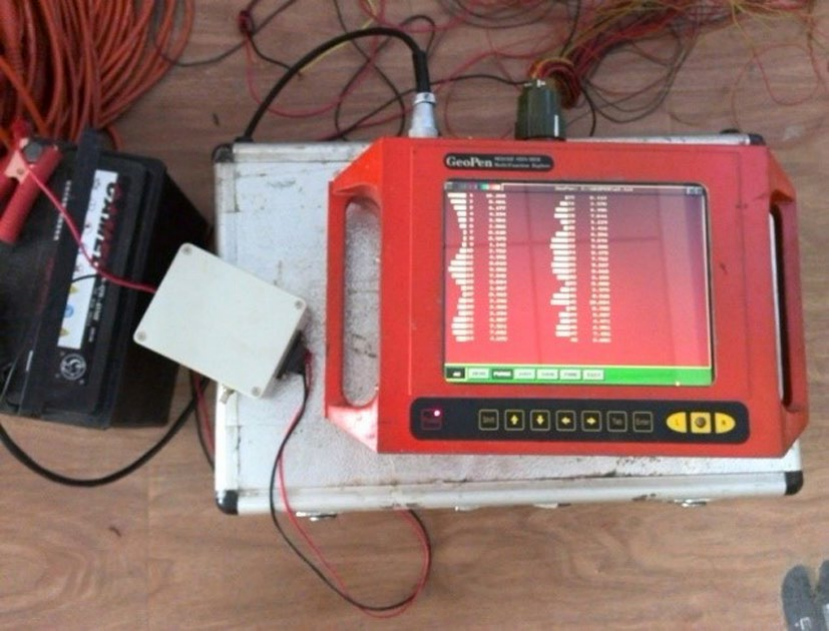
Se2432 parallel electric method instrument.

The mainframe can control the operation of the monitoring system. The possible leachate points and their pollution range are determined by collecting data. The system mainly includes mainframe and its software system, power supply, etc., as shown in Fig. 3.

### Leachate device

Place 4 leakage bottles above the tank. No.1 and No.4 bottled water are used to simulate the leakage liquid formed by the direct infiltration of rainwater in slag through geomembrane and as a reference. Because Cl^-1^ is a typical pollutant in the landfill. No. 2 bottle containing 20 g/L NaCl solution is used to simulate inorganic salt leakage in urban life. No. 3 bottle containing 20 ml/L ethanol solution is used to simulate the leakage liquid containing a large amount of organic matter in municipal solid waste. The characteristics of leachate have been summarized in Table1.

**Table 1:** The characteristics of leachate.

No	Solution	Capacity	Concentration
1	Water	6L	–
2	NaCl	6L	20 g/L
3	Ethyl alcohol	6L	20 ml/L
4	Water	6L	–

Before the experiment, configure four solutions, close the injection, use an electric meter to check the conductivity of each measuring point. After each measuring point has no open circuit, supply power to the soil layer through the mainframe to measure the background electric field of the soil. Then open the injection, adjust the flow rate, release the solution at a fixed flow rate, record the soil electric field in the process of leakage every half an hour, collect the potential values of each measuring point, process the data through the potentiometry and potential difference method, and form the relevant potential horizontal profile and longitudinal section of the soil.

### Principle of potentiometric detection technology

When there are leakage points in the landfill, power is supplied to the landfill, and the current forms a current loop through the geomembrane. If there are n (n = 1,2,3…) leakage points in the geomembrane, the power supply current is I, and the artificial electric field will form a leakage electric field at the leakage point, which can be used as a point power supply.

1 $$I = \int dI = \int j \cdot dS$$

where I is the current intensity, j is the current density vector, and S is the area passing through the current.

When there are n leakage points, I will be shunted. If a leakage point is regarded as a finite surface, the current intensity I as:

2 $$I = {I_1} + {I_2} + \cdot \cdot \cdot + {I_{\text{n}}} = \sum\limits_{i = 1}^n {\int_{S_i} {jdS} }$$

Generally, the power supply current field of landfill site will be affected by the formation medium structure. It is assumed that the formation medium structure is composed of three layers, each layer has uniform properties and stable conductivity, and the layers from top to bottom are: landfill layer, with resistivity of ρ1. The saturated leakage liquid layer above the geomembrane has a resistivity of ρ2. The clay layer under the geomembrane has a resistivity of ρ3. The electrode C1 is arranged in the garbage layer for power supply, and the electrode C2 is arranged at the lower part of the geomembrane away from the electrode system area. The electrode C2 can be regarded as a far pole.

Because of the ρ1 > ρ2, the conductivity of the saturated leakage liquid layer at the upper part of the geomembrane is better than that of the landfill layer, so that there is almost no reflected current between the ρ1 layer and the ρ2 layer, that is, the current generated by the power supply electrode C1 is all transmitted to the ρ2 layer. Because of the ρ3 > ρ2, it can be considered that the interface between ρ2 layer and ρ3 layer has both a reflection current, and a transmission current through the leakage point. The potential generated at the detection electrode P1 under the geomembrane is formed by the action of transmission current. The total potential of point P1 is obtained by the superposition of the potential of point power supply passing through n leakage points at P1.

3 $${U_{P1}} = \sum\limits_{i = 1}^n {\frac{{{I_i}{\rho_3}}}{{2\pi {{\text{r}}_{iP1}}}}}$$

### Parallel potential difference method

The test adopts pole–pole arrangement, and the calculation formula of apparent resistivity is as follows:

4 $$\rho = 2\pi {\text{aR}}$$

where ρ is apparent resistivity; a is the distance between electrodes C1 and P1; R is measuring resistivity.

When there are loopholes in the geomembrane of the landfill, the leakage liquid will gradually penetrate into the soil layer under the geomembrane through the loopholes, resulting in the change of the conductivity of the soil layer under the geomembrane. The pole-pole acquisition mode of Se2432 parallel electrical instrument is used to obtain the original data (potential difference) of each measuring point on the grid. After current normalization, the apparent resistivity of the soil layer is obtained. The electrical properties of different depths of the soil layer can be obtained by inversion of the apparent resistivity data of the soil layer, so as to determine the occurrence point and distribution range of leakage.

**Figure 4:**
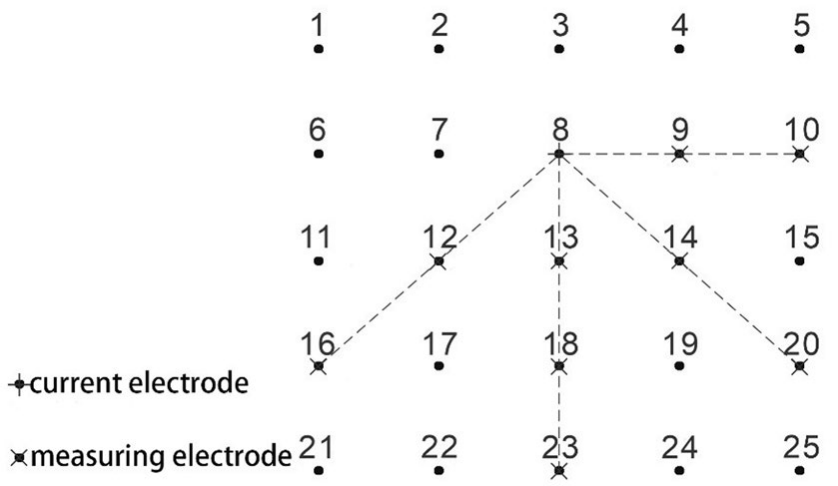
Schematic diagram of cross-diagonal measurement method.

The monitoring grid is 5 × 5. The spacing between measuring points is 0.08 m. The measurement method adopted by Se2432 parallel electric method instrument is cross diagonal measurement method. Figure 4 shows that it only needs to measure the potential values on the measuring points on the horizontal, vertical and 45° diagonal lines.

### Theoretical calculation of test model

#### Theoretical results of 10 × 10 grid monitoring

According to the experimental model and statistical data, the resistivity of the clay layer under the geomembrane is assumed ρ = 10 Ω· m, the resistivity ratio of tap water, NaCl solution and ethanol solution after penetrating into the soil layer ρ_No.1_:ρ_No.2_:ρ_No.3_ = 5:3:10. If the four leakage points set by the model are regarded as four conductive resistors, the ratio of the current passing through the four leakage points is I_No. 1_:I_No. 2_:I_No. 3_:I_No. 4_ = 6:10:3:6.

**Figure 5:**
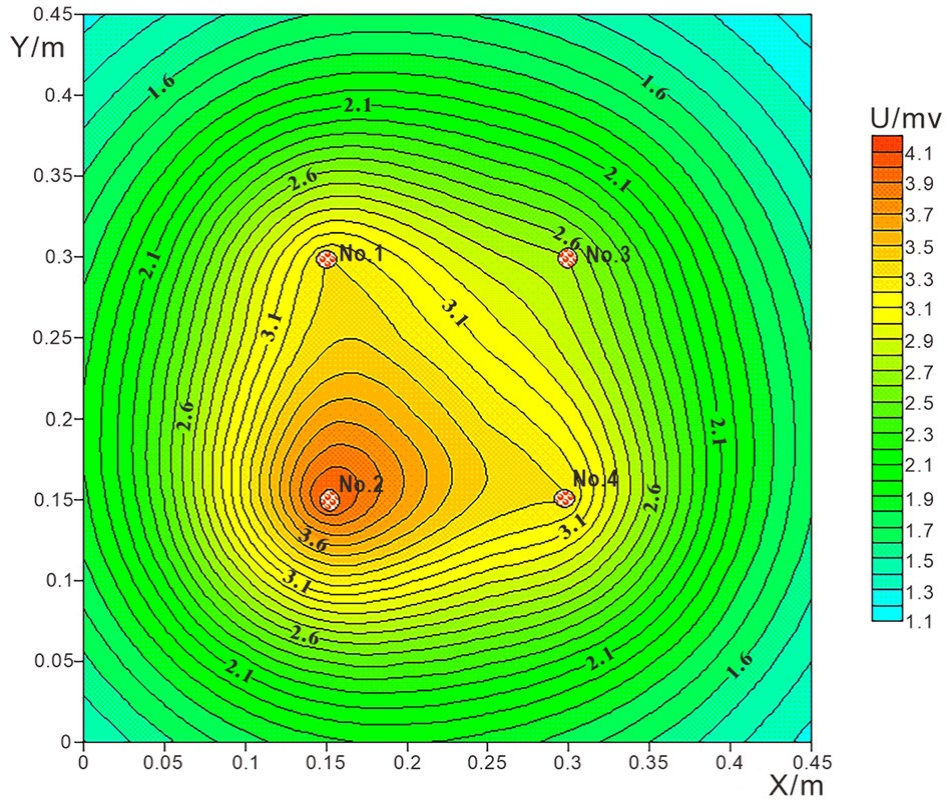
10 × 10 Grid theory detection potential contour map.

The calculation model is 10 × 10 grid, and the spacing of measuring points is 0.05 m. The potential value on each measuring point is calculated according to Eq. 3, and the obtained data is processed with surfer software to obtain the potential contour map, as shown in Fig. 5. Among them, points 1, 2, 3 and 4 are the leakage positions of water, NaCl solution, ethanol solution and water respectively, and the spacing between leakage points is 0.15 m.

Figure 5 shows that the leakage fields formed by the four kinds of leaking liquids interfere with each other from the theoretical calculation results. The leachate current at point 2 is larger, the high potential closed loop is obvious, and its center corresponds to the leakage center. The reason for this is that the NaCl solution contains conductive particles that increase the conductivity of the leak point. Point 1 and 4 are the same as water, and the leakage electric field is almost the same. Its closed loop is obvious, and the high potential center also corresponds to their leakage position. There is almost no closed loop effect at point 3 under the influence of 1, 2 and 4. The results show that the leakage field formed by high resistance leakage liquid is not easy to be detected by potentiometric detection, and low resistance leakage is suitable to be detected by potentiometric detection.

#### Theoretical results of 12 × 12 grid monitoring

**Figure 6:**
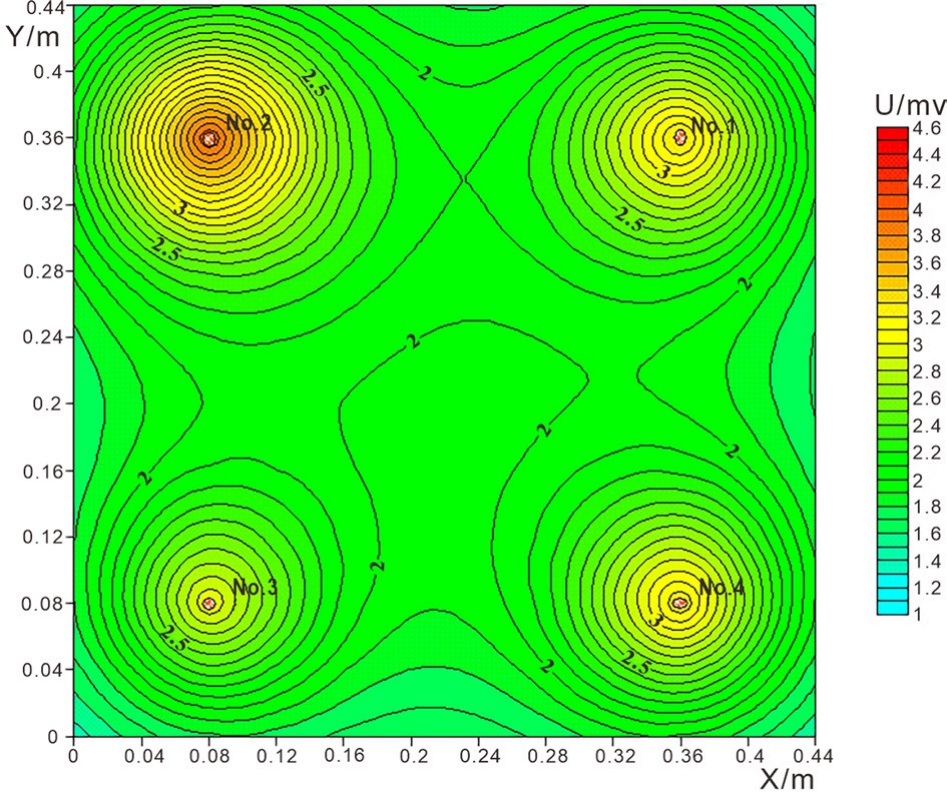
12 × 12 Grid theory detection potential contour map.

The resistivity of the clay layer under the geomembrane is assumed ρ = 10Ω·m. In consideration of the mutual influence between the leachate and appropriately reduce its influence effect, the resistivity ratio of water, NaCl solution, and ethanol solution after penetrating into the soil layer is set as ρ_No.1_:ρ_No.2_:ρ_No.3_ = 20:15:24, the ratio of the current passing through the four leakage points is I_No.1_:I_No.2_:I_No.3_:I_No.4_ = 6:8:5:6. And adjust the distance between the two points to 0.28 m. 12 × 12 grid was used for detection, and the spacing of detection points is 0.04 m. Calculate the potential value of each detection point according to Eq. 3, and use Surfer to obtain the detection contour map of four kinds of leakage, as shown in Fig. 6.

Theoretical calculation results show that when the distance between the leakage points is large and the distance between the detection points is small, the potentiometric method can detect the leakage position of various leachates well. At the same time, the diffusion range of different leachates in the same plane is roughly the same, and they all gradually diffuse outward from the center of the leakage point, and the potential value gradually decrease. Point 2 has the largest potential closed loop range, which also has a certain impact on the leakage points of adjacent points 1 and 3. Point 1 and point 4 are water leakage. Affected by different leakage liquids, the leakage electric field of the two same leakage liquids is obviously different. The potential closed loop range of point 1 is larger than that of point 4. Point 3 is the leakage of ethanol solution. Because its resistance is the largest, the range of potential closed loop is the smallest.

**Figure 7:**
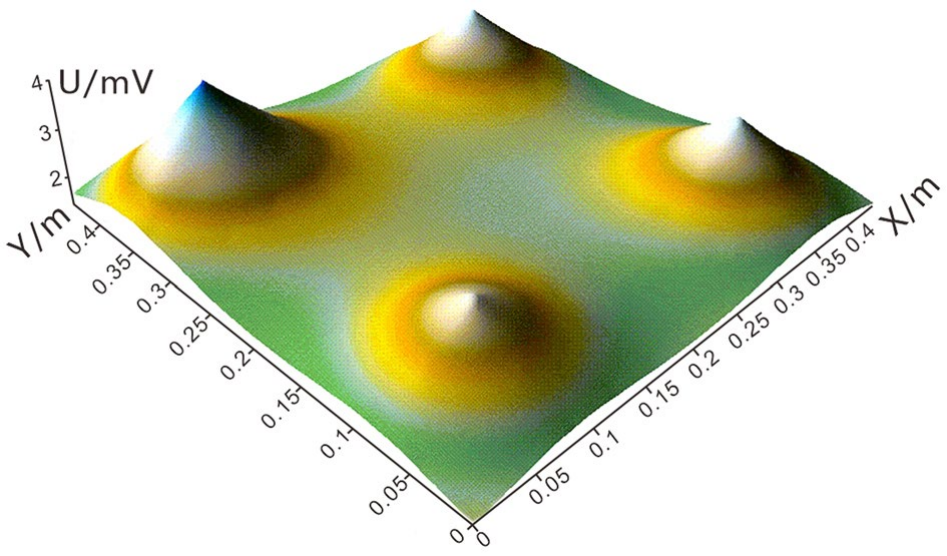
12 × 12 Grid theory detects potential 3d view.

Figure 7 shows that the leakage fields around the leachates are funnel-shaped, and its size is related to the type of leachate. Therefore, different network density should be designed for different types of leakage liquid, so as to use the most economical scheme to detect the leakage point.

### Interpretation and discussion of results

#### Laboratory simulation experiment research

**Figure 8:**
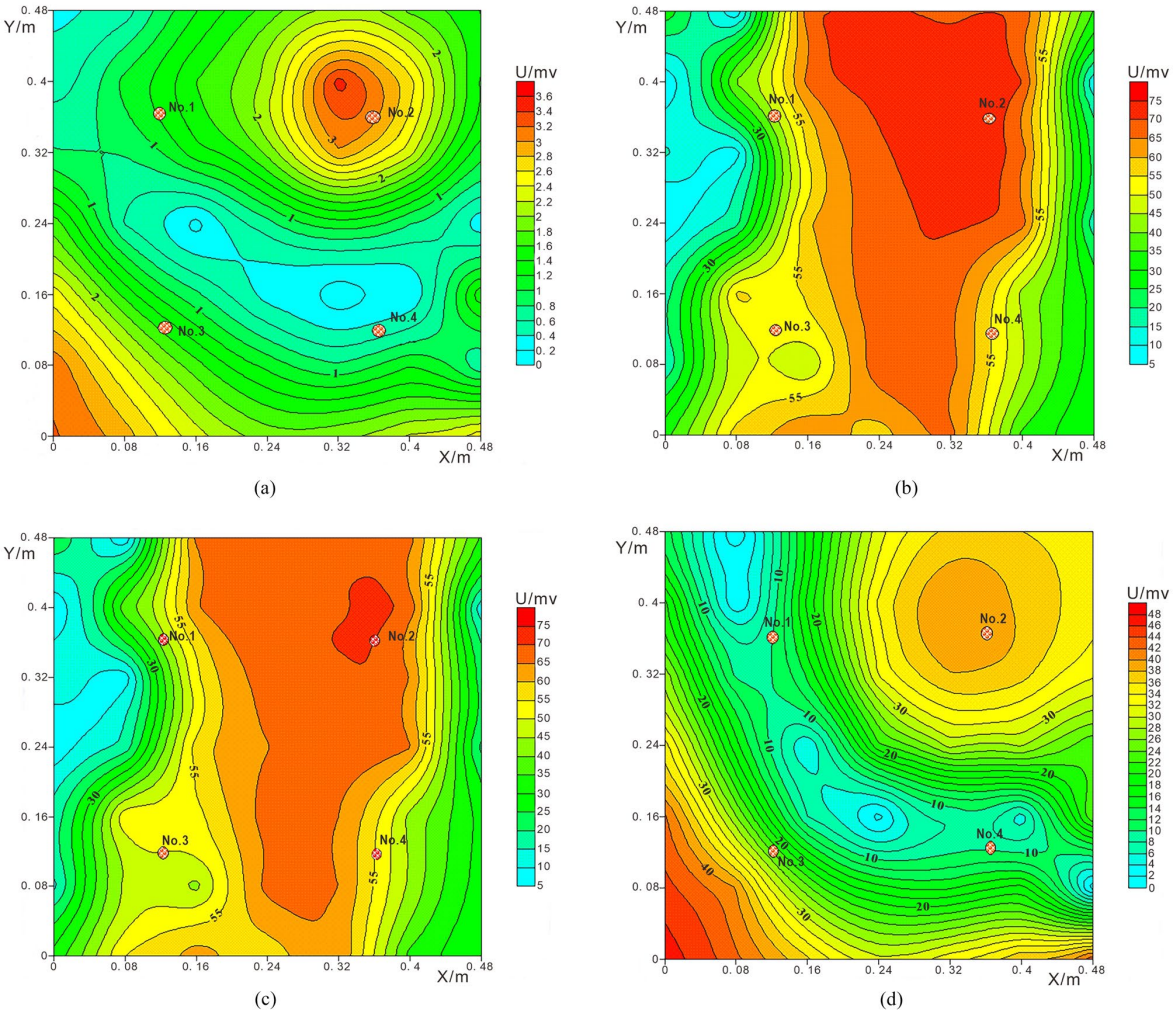
Leakage field potential diagram of soil layer: (**a**) Background electric field of soil layer; (**b**) Potential distribution of soil layer after 1 h of leakage; (**c**) Potential distribution of soil layer after 2 h of leakage; (**d**) Potential distribution of soil layer after closing the injection tube for 30 min.

Figure 8a shows the background electric field potential of soil layer. The four injection pipes are opened at the same time and adjusted to the same flow rate. Under the condition of continuous leakage, monitor the leakage field potential at an interval of 1 h. Figure 8b shows the leakage electric field potential value for 1 h. Reduce the injection pipes flow rate to 1/2 of the initial value. Figure 8c shows the monitoring results of 2 h soil layer leakage field potential. Figure 8d shows the soil leakage field potential monitored after 30 min of sealing the injection pipes.

Figure 8a shows that the background potential contour of the experimental soil layer is at a lower value. Few current lines pass through the monitoring area. A dense closed potential circle of high potential value is formed at point 2. The current flow at point 2 is greater than the other points 1, 3 and 4. The analysis result may be that in the process of watering and compaction, the clay layer under the geomembrane is not uniform, and the compaction degree of the soil layer is different, resulting in different potential values ​​obtained by monitoring. The permeability at point 2 is better than other points, so when the flow rate of the leakage liquid is large, the leakage liquid under the geomembrane gathers near point 2 and spreads out around. After the clay is watered and compacted, the soil compaction is small and the pore water content is large, resulting in a high potential abnormal area in the lower left corner of point 3.

Point 2 forms a closed loop of anomaly potential contour much higher than the background electric field, while the value of potential contour coil at leakage point 3 is lower than the surrounding value. It can be analyzed that positions 2 and 3 are leakage points. The leachate at point 2 is a high concentration NaCl solution containing more conductive particles, which will reduce the resistivity of the soil layer under the geomembrane at point 2. Thus, the passing current is increased to form a high potential closed loop. The leachate at point 3 is ethanol solution, which will increase the resistivity of the soil layer under the geomembrane at point 3. So as to reduce the passing current and form a low potential closed loop. Figure 8b shows that the potential contour is consistent with the influence of NaCl solution and ethanol solution on the soil layer under the geomembrane. It can be concluded that point 2 and point 3 are leakage points. The electric field formed after water leakage at point 1 and point 4 cannot clearly distinguish the leakage points.

During the monitoring process, the leachate was continuously released from the injection pipe, and the results reflected the dynamic characteristics. Figure 8b shows the phenomenon that the leachate from point 1 and point 4 aggregates around point 2, which is consistent with the inference of better permeability at point 2. Figure 8b,c show that when the flow rate of the leachate is changed and the flow rate of the injection pipe is reduced, the high-potential region of the entire electric field is reduced. Under the influence of gravity, the leachate will migrate longitudinally, and the closed-loop abnormally high-potential regions and abnormally low-potential regions at points 2 and 3 also decrease.

Compared with the surrounding potential contours, the difference is more obvious. Figure 8d shows that when the injection pipe stops leaking for a period of time, the leachate migrates longitudinally along the leakage point. At this time, the electric field of the soil layer is similar to the original background electric field, but the potential value is higher than the background electric field, indicating that the leachate is stagnant in the pores of the soil layer, it is the result of changing the electrical properties of the soil layer. The parallel potential method can collect the potential value of each point in the field at one time, which provides a basis for real-time monitoring of landfill leachate.

**Figure 9:**
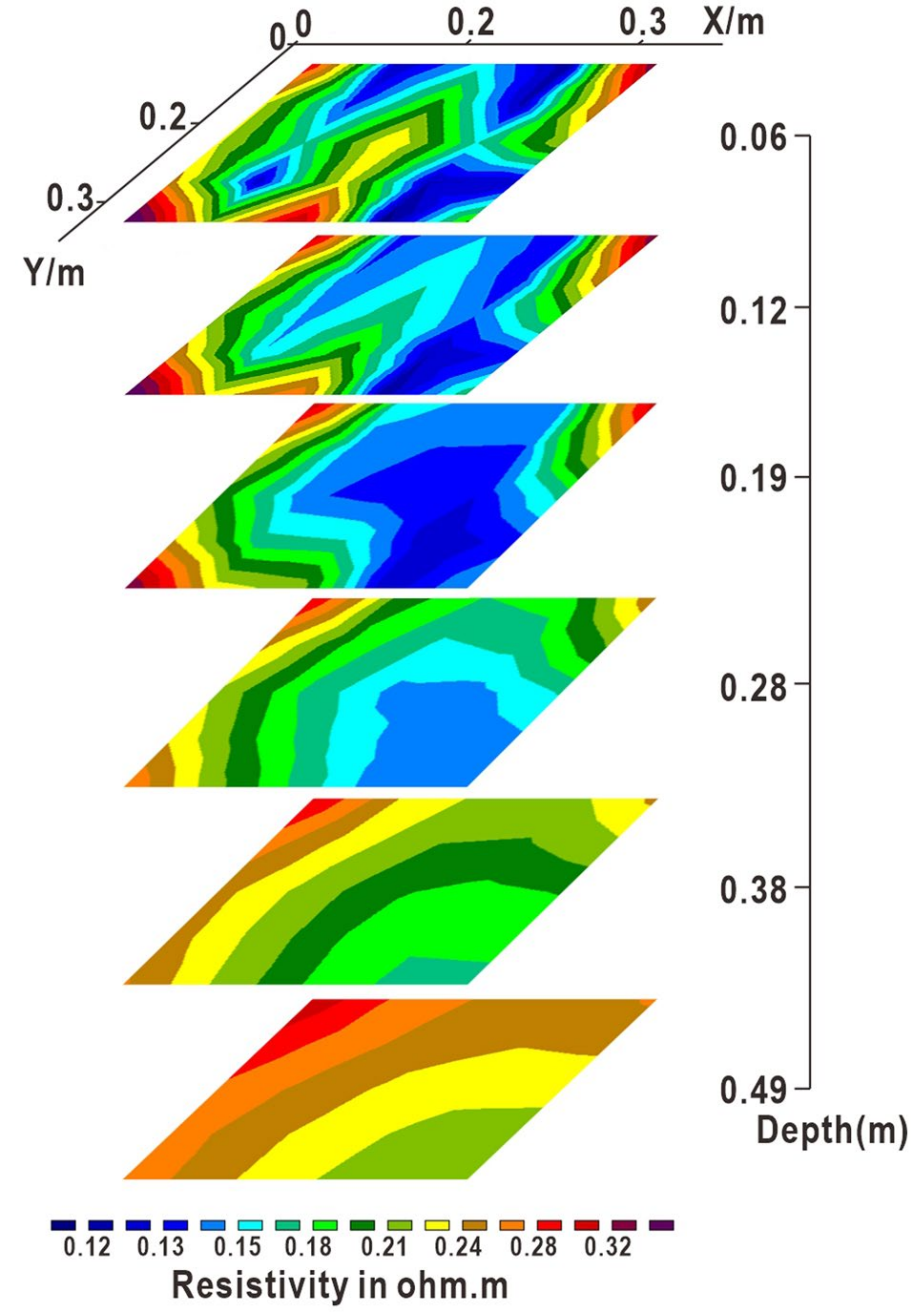
Inversion map of plane section at different depths.

Figure 9 shows the inversion results of the horizontal section of the experimental model. The blue area corresponds to the distribution range of the low resistance anomaly. There are no jump or distortion points in the profile. The resistivity in the longitudinal direction basically shows a change from low to high. The upper layer seepage liquid migrates, and the bottom soil layer is characterized by low humidity and high resistivity. The low-resistance areas formed by the leakage of NaCl solution are widely distributed in the horizontal section. The distribution range is 0–0.28 m, and the migration scale of the leakage liquid can be clearly seen. The morphological characteristics of water leakage in different parts are basically the same. The distribution range is 0–0.18 m. The leakage of ethanol solution is only reflected at 0–0.06 m, and the distribution range is the smallest at the same depth. The ethanol solution also had the slowest migration rate.

**Figure 10:**
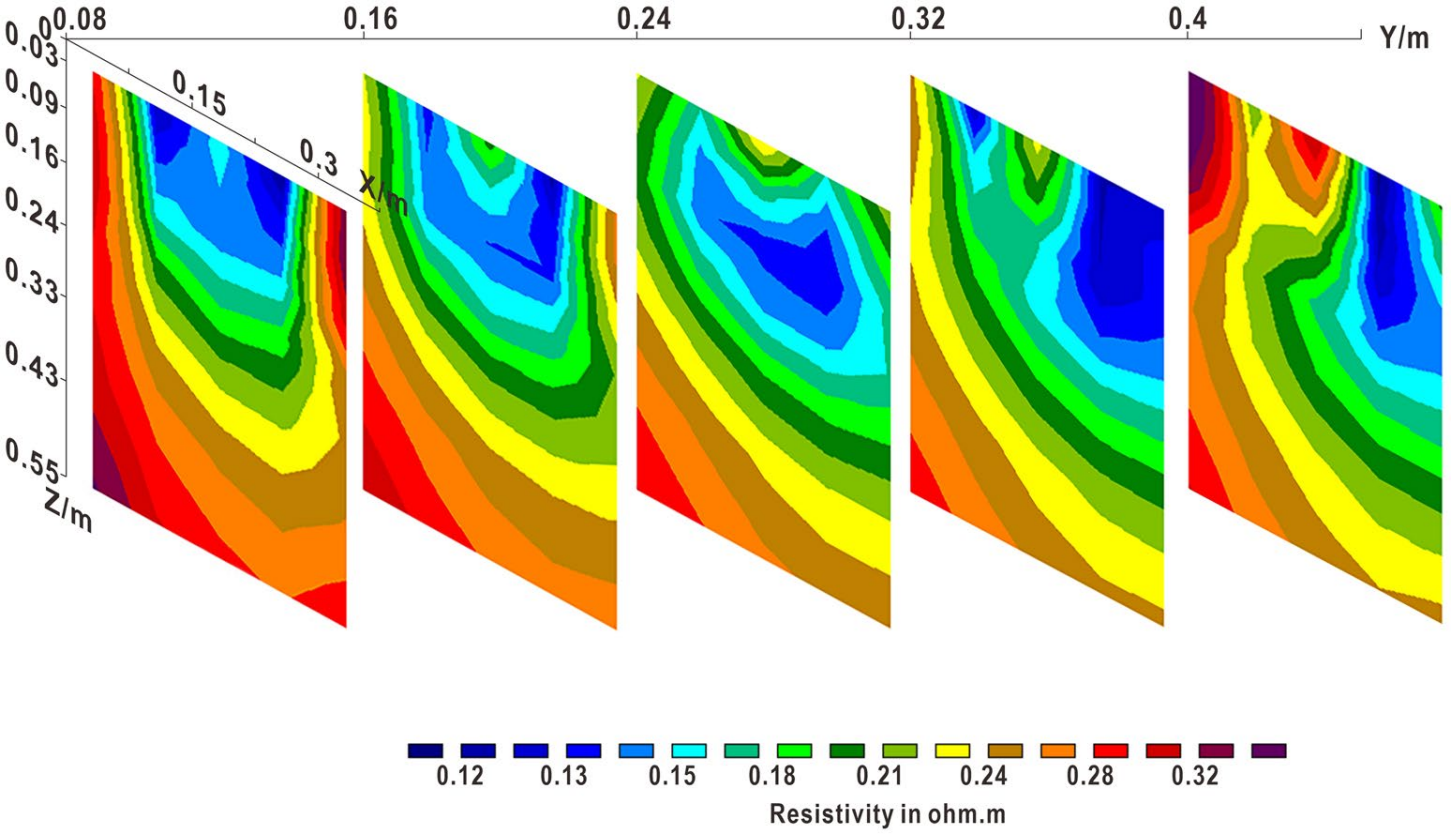
X–Z longitudinal section on different Y axes.

Figure 10 shows the inversion results of the X–Z longitudinal section of the test model. The two apparent resistivity profiles at Y = 0.24 m and Y = 0.32 m show that there is no low-resistance area in the shallow layer on the soil layer, indicating that the geomembrane in this area is not damaged. The low resistance zone in the middle is caused by the lateral migration of leakage fluid. The low-resistance anomaly area at the top of the profile can be judged as a leak point or formed by the migration of nearby leachate. Combined with the horizontal section, the leakage depth is similar, and the lateral migration speed of leachate is faster than the longitudinal migration speed. Four leak points can be distinguished, delineating the general location of the leak.

#### Physical model experiment

The potential value of each electrode was monitored after 2 h of leakage, and the resistivity profiles at different positions were obtained by the potential difference method.

**Figure 11:**
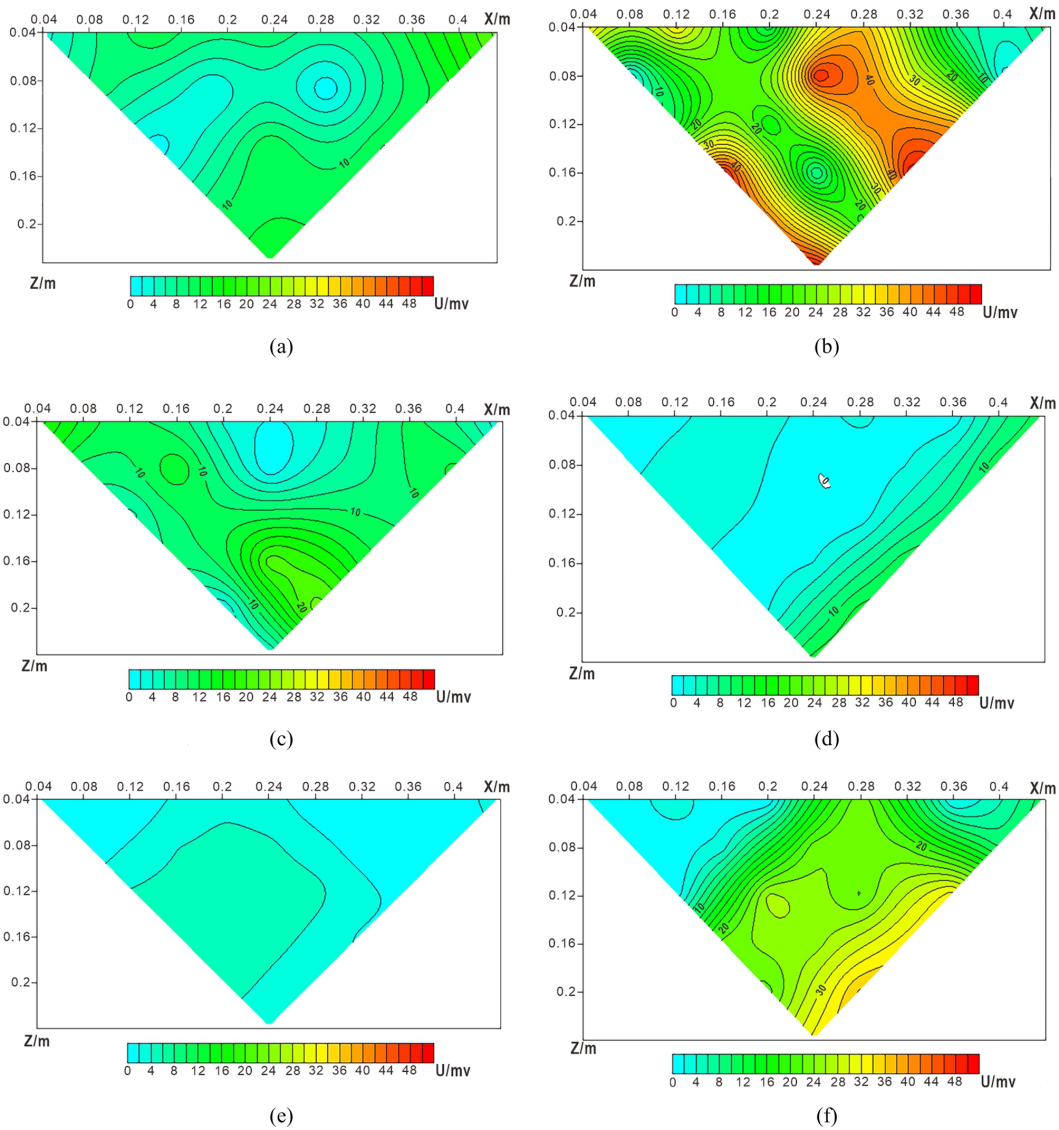
Electrical resistivity tomograms of profile: (**a**) Resistivity of the slitting profile Y = 0; (**b**) Resistivity of the slitting profile Y = 0.08; (**c**) Resistivity of the slitting profile Y = 0.16; (**d**) Resistivity of the slitting profile Y = 0.24; (**e**) Resistivity of the slitting profile Y = 0.32; (**f**) Resistivity of the slitting profile Y = 0.4.

It can be seen from Fig. 11 that the potential difference method can monitor the leakage of leachate in different directions. The morphological features of the plume formed by the downward migration of the leak point are approximately funnel-shaped in longitudinal section. The affected area of ​​the soil layer can be obtained in time. Figure 11b shows that the potential difference at the monitoring point is very different on both sides. After 2 h of leakage, a large amount of leakage liquid exists in the soil layer. When the water content in the soil layer increases, the diffusion rate of the ethanol solution increases, showing high resistance characteristics. At the same time, due to the action of gravity, there is a lot of vertical migration, and the potential value changes greatly. The profile clearly shows that the distribution area of ​​high potential difference is large, and the distribution of low potential is small. Figure 11c shows that since the migration rate of leachate in the horizontal direction is greater than that in the vertical direction, the potential difference of the monitoring point in the middle region is smaller, and a closed region of a high-potential circle is formed in the middle. The difference between the two results in a smaller potential difference area. Figure 11d shows that almost all the low-potential areas on the monitoring point are on the left side, because the leakage rate of NaCl solution in the horizontal direction is similar to that in the vertical direction under the condition of good soil compaction. At this time, a large number of conductive particles are contained, resulting in a large high-potential region. The difference between the two forms a large area of ​​low potential difference on the left. This is in good agreement with the lower resistance characteristics of the NaCl solution. Figure 11e shows that the two low-resistance regions correspond to the two leakage centers. The low potential difference region is formed by migration around the leak point. The migration speed in the horizontal direction is similar to that in the vertical direction, and the water migration speed on the left is slower than that of the sodium chloride solution on the right. Figure 11e,f show that the monitoring results are the same, but the resulting potential difference is also increased. This is affected by the distance between the monitoring point and the leak point. When the monitored point and the leakage point are located on the same section, the soil layer is the most severely affected area by leakage. Through the change of the potential difference, the leakage range and the location of the leakage point can be better judged.

## Conclusion

Monitoring for potential leaks is necessary. Soil and groundwater sampling through borehole surveys is expensive and discrete single-point data that is difficult to spatially interpret. In this paper, according to the research status of landfills, an indoor physical model test of multiple leachate and multiple leakage points is designed and made. Firstly, the experimental model is theoretically solved, and then the leakage process of the three leachates is monitored in real time. The calculation results were calculated using the potential method and the potential difference method, and the following conclusions were drawn:

In order to achieve better monitoring accuracy and highlight the migration range of leachate, especially for landfills in areas with heavy rainfall and rainy season climate, the density of electrode grids should be increased because a large amount of precipitation further affects the migration of leachate. In this way, when using the potential difference method to solve, the effective area of ​​the longitudinal section will increase to reflect the leakage migration when edge leakage occurs. Theoretical calculations show that the potential method and the three-dimensional inversion program can well reflect the location, number and distribution of leaks at different depths. For more types of leachate, further experiments will be carried out at a later stage to obtain more comprehensive monitoring results.

The application of test results is often limited due to many uncontrollable factors that may affect the accuracy of field testing. The physical model produced can monitor the leachate migration law at varying flow rates. The properties of the clay layer under the geomembrane have a significant effect on the law of leakage and migration. During the actual construction of the landfill, the clay layer under the geomembrane should be as uniform as possible. When there are few abnormal areas of the background electric field in the soil layer, the monitoring point data is more accurate in judging the leakage point. The migration of leachate on the horizontal plane and each longitudinal section can also be obtained, with better sensitivity and a better description of the plume range of the leakage point. The difference between high resistance and low resistance in the abnormal area is convenient for the selection of different types of pollutants in the later stage.

In the early stage of leakage, the leachate mainly migrates laterally. Longitudinal migration increases when soil moisture content increases. The water pressure of the leachate has a great influence on the leakage process. When the water pressure of the leachate is large, the measurement results are not easy to distinguish the location of different leakage points. When the hydraulic pressure is low, the location of the leak point in the monitoring results will become more obvious. This method is an effective way for leachate monitoring.

## Data Availability

The datasets generated during the current study are available from the corresponding author on reasonable request.
